# CLAHE-CapsNet: Efficient retina optical coherence tomography classification using capsule networks with contrast limited adaptive histogram equalization

**DOI:** 10.1371/journal.pone.0288663

**Published:** 2023-11-30

**Authors:** Michael Opoku, Benjamin Asubam Weyori, Adebayo Felix Adekoya, Kwabena Adu

**Affiliations:** Department of Computer Science and Informatics, University of Energy and Natural Resource, Sunyani, Ghana; University of Manitoba, CANADA

## Abstract

Manual detection of eye diseases using retina Optical Coherence Tomography (OCT) images by Ophthalmologists is time consuming, prone to errors and tedious. Previous researchers have developed a computer aided system using deep learning-based convolutional neural networks (CNNs) to aid in faster detection of the retina diseases. However, these methods find it difficult to achieve better classification performance due to noise in the OCT image. Moreover, the pooling operations in CNN reduce resolution of the image that limits the performance of the model. The contributions of the paper are in two folds. Firstly, this paper makes a comprehensive literature review to establish current-state-of-act methods successfully implemented in retina OCT image classifications. Additionally, this paper proposes a capsule network coupled with contrast limited adaptive histogram equalization (CLAHE-CapsNet) for retina OCT image classification. The CLAHE was implemented as layers to minimize the noise in the retina image for better performance of the model. A three-layer convolutional capsule network was designed with carefully chosen hyperparameters. The dataset used for this study was presented by University of California San Diego (UCSD). The dataset consists of 84,495 X-Ray images (JPEG) and 4 categories (NORMAL, CNV, DME, and DRUSEN). The images went through a grading system consisting of multiple layers of trained graders of expertise for verification and correction of image labels. Evaluation experiments were conducted and comparison of results was done with state-of-the-art models to find out the best performing model. The evaluation metrics; accuracy, sensitivity, precision, specificity, and AUC are used to determine the performance of the models. The evaluation results show that the proposed model achieves the best performing model of accuracies of 97.7%, 99.5%, and 99.3% on overall accuracy (OA), overall sensitivity (OS), and overall precision (OP), respectively. The results obtained indicate that the proposed model can be adopted and implemented to help ophthalmologists in detecting retina OCT diseases.

## 1 Introduction

The eye is one of the most important organs in the human body that ensures one’s ability to see. The visual impairment of eye can lead to total blindness as it directly affects one’s vision if attention is not given in due time. The ripple effect can indirectly affect one’s mobility, sense of self, independency, ability to undertake other basic activities required even on daily bases. A statistics presented in 2019 by WHO still indicated that about 2.2 billion people have been affected with vision impairment and blindness which about 80% could have been avoided if detected during early stage [1, 2]. Therefore, performing medical diagnosing and detecting possible eye disease early enough so that a proper remedy can be recommended to provide an effective solution is always essential to support the work of the ophthalmologists especially in real time. This has necessitated many research work to focus on detecting possible type and causes of most visual impairment which macula disease has been identified by many studies as major causes [3–5]. Macula is located in the middle part of the retina which is exclusively in charge of controlling and ensuring the central vision. The retina however represents a thin tissue serving as a lining to the inside back layer of the eye which receives and transforms light signals through the optic nerves to the section of the brain where vision occurs [6]. It is considered as the most important portion of the eye. The retina can be divided into several parts such as the receptor layer, pigment epithelium, cell layer, internal limiting membrane, external limiting membrane, and vitreous body. The retina is responsible for receiving and converting light waves into neural signals which are passed on to the brain for visual recognition. This means it provides the brain with details on what has been perceived and provides the ability to differentiate between objects, colors and many other things. Therefore, when the section of the retina called macula is affected by a disease, then it affects the actual functionality of the retina hence the eye might have issues with visualization. The disease is usually caused by different pathological factors which causes the macula to gradually deteriorate over a long period of time. In this case, a perceived image is not well received and details are also not correctly captured which should have been the basic responsibility of the macula in the retina. Once affected, if treatment is not initiated on time, then it can lead to irreversible vision loss which is very common in adults who are over 60 years [4]. At early stages, the disease may not affect vision but one is likely to experience diminished abilities to perceive objects clearly in low light. If not detected at early stages so treatment can be initiated, then the affected person might experience blurred vision with time until the central macula loses complete vision [7]. This could automatically lead to complete blindness but where some aspect of the retina still functions then the person is likely to experience peripheral vision cannot be compared to central vision in any sense. The current therapy can control progression of the disease and in some cases, possible reversal of loss vision [8–10]. Macula disease in general has been considered as an incurable disease but can be managed if detected at early stages hence the many research interests in this domain to improve early detection. Though the actual cause is still not visible to science, there are other contributing factors including some hereditary, environmental factors (poor dieting, smoking, continues exposure to sun rays), malfunctioning of the body mechanism which can lead to deterioration of the cells of the macula located in the central part of the retina. According to Fang et al., in 2019, it was explained in their article the biggest risk factor that can cause macular disease is age [3]. This has led to three possible outcomes thus the age-related macular degeneration (AMD), choroidal neovascularization (CNV), and diabetic macular edema (DME). For ophthalmologists, effective treatment can only commence if the extent of infection can only be concluded whether it is one of the three expected outcomes. The study attempts building a deep learning technique known as capsule network with contrast limited adaptive histogram equalization in the field of medical science to automatically detect and perform classification on retinal OCT imagery of macular diseases. The classification would establish whether a selected image belong to Drusen,CNV, DME and Normal class of images. If the model performs extremely well then it would bring a promising revolution to the field of medical science and a significant solution that can impact the clinical practice of eye disease diagnoses.

There are two fundamental types of age-related macular degeneration, which are dry and wet AMD. These names are derived based on the physical changes affecting the macula in the retina which one is likely to experience when affected by AMD. Affected persons with AMD usually begin with dry form which may advance to intermediate stage and final stage which is known as geographic atrophy. This might be detected by an ophthalmologist according to imaging taken during a dilated pupil examination. The dry AMD can lead to severe metabolic activities produced by the photoreceptors found in the retina which might be accumulated behind the macula to form drusen. This drusen might increase in size over time the waste removal system mechanism is unable to remove such fragments from people with AMD. A typical characteristic that might be exhibited by AMD is the presence of drusen, which represent asymptomatic deposition of extracellular fragment found in between the retinal pigment epithelium (RPE) and the inner collagenous layer of Bruch’s membrane [3]. The presence of drusen is not the main cause of AMD but large numbers could indicate dry AMD. The drusen comprises of fats and proteins which therefore makes it difficult for absorption of nutrients sent to photoreceptors by the RPE. Once access to required nutrients is prevented, the photoreceptors gradually die out for lack of nutrients and its effect reduces visual acuity, loss of contrast and color vision sensitivity. A blank space called the dead zone in the central visual part of the retina is created as the photoreceptors die out beyond a certain threshold which makes it impossible to perceive and interpret details as required. The dangers involved is large drusen can shift the normal position of the photoreceptors which can lead to a condition that causes straight lines to be seen as wavy lines. However, up to 15% of patients with dry AMD may gradually metamorphose into wet AMD in an advanced stage. Others may also experience both wet and dry AMD at an advanced stage. However, when AMD develops into the advanced stage, where the photoreceptors of certain areas of the retina are considered dead and those areas have also lost pigment, such abrasion is considered as irreversible vision loss. When AMD is at this stage it is known as CNV. DME is a complication resulting from built-up fluid in the form of cysts and exudates in the macula which represents the central portion of the retina. This fluid is as a result of damaged retinal blood vessels which causes retinal thickening. This developed complication is referred to as diabetic retinopathy which can lead to blindness in people with severe diabetes. This makes it an interesting area for researchers to establish possible detection strategies that can best help ophthalmologists to detect macular diseases. DME is of two types namely Non-Central which involves diabetic macular edema in mild state and Central which involves diabetic macular edema in severe state. The risk factors associated to DME include diabetes, kidney disease, excessively high blood pressure, high levels of fat in the blood, fluid retention, pregnancy etc. Optical coherence tomography (OCT) is a simple imaging technique that employs reflected light wave to capture cross-section pictures of the retina which is located at the back of the eye. The OCT makes it possible for the ophthalmologist to see and analyze each distinctive layer of the retina and measure their thickness to helps in diagnosing of macula diseases and diabetes related retinopathy diseases [11, 12]. The OCT is accepted standard for many ophthalmologists in clinical trials and clinical practices for diagnosing the various progression stages of the macula disease [13, 14]. The modern spectral-domain OCT permits non-invasive 3D visualization at high speed resolution for retina analysis. This means the modern OCT can accurately provide a detailed 3D shape and the level of the drusen as well as the rate of change from early stages through the intermediate to the advanced stage over time. It may also provide signs of neovascularization at advance stage and even predict the atrophic areas for further attention [15–18]. The analysis is usually done manually at each cross-section of the OCT volume and the final decision for a particular diagnosis is specifically made by the ophthalmologist to determine the type of disease. As an ophthalmologist, one needs attention mechanism for visual perception system in other to focus on the important areas for OCT image [19]. According to Fang et al., 2019 [3], it is a mechanism needed to focus and detect the salient areas required from the presented OCT image for better analyses instead of processing the entire image. The saliency in OCT image is considered the lesions which is the area that require concentration for proper clinical diagnosis. Nonetheless, the manual process requires review of multiple OCT scans volumes which might consume a lot of time. Moreover it might be susceptible to errors in an attempt to extract the required distinguishing features to diagnose the disease. In some cases, it might require the attention of experts whose input can also impact subjectively on the results. Approximately 30 million OCT scans are performed each year, and analyzing or interpreting these images takes significant time [20]. As a result it has become necessary to employ computer assisted OCT image analyzer which ensures high quality analysis to effectively detect the presence of the macula disease within the shortest period of time. Proper implementation might release ophthalmologists, the burden of conducting multiple screening of OCT volumes. Moreover, the results from the computer assisted OCT image analysis can quickly help the ophthalmologist to make effective decisions about the progression rate of the macula disease. Although performance of these computer vision techniques could to some extent be very promising, their reliability cannot always be 100% efficient as these algorithms have tendency of failing if not properly implemented [21, 22]. The performance of a model in classification of OCT volume is of essence. However, the effectiveness and efficiency of any model depends on its ability to classify data accurately with minimum error. Depending on the applied technique and methods used, the output performance for one model might always outperform the other. With the introduction of deep learning techniques in computer vision which could process images and ensure segmentation of layers, a lot of attention were specifically diverted into retinal OCT image analysis [23, 24] which majority were implemented using convolutional neural network (CNN) [24–26]. The CNN is effective however, it has limitations which can affect the performance of the model. CNN loses some important features of the image due to the pooling operation which affects the resolution of the image [27]. Furthermore, the CNN is more susceptible to adversarial attacks like the pixel perturbations which can lead to wrong prediction [28, 29]. The CNN in implementation is not able to recognize pose, texture, deformations or part of an image [30]. In an attempt to resolve this CNN problem, Sabour et al., 2017 [30] proposed the Capsule Network (CapsNet) with dynamic routing algorithm which gained a lot of popularity as a result of its performance. This paper seeks to implement a capsule network coupled with contrast limited adaptive histogram equalization (CLAHE-CapsNet) on retina OCT image dataset and compare the efficiency of the model with other state-of-the-act models for performance evaluation.

The contributions of the study are summarized as follows;

- This paper proposes a capsule network with contrast limited adaptive histogram equalization (CLAHE-CapsNet) for retina OCT image classification.
- The study also makes a comprehensive literature review to establish current-state-of-act methods successfully implemented in retina OCT image classifications.
- The study compares the proposed model with the original capsule network for performance efficiency.
- Four-class retina OCT image dataset was used for training and testing the proposed capsule framework. The evaluation results are compared with other deep learning convolutional neural networks-based methods which our proposed method obtained the better accuracy technique for detecting retina OCT-base eye disease.

## 2 Related works

Many researchers started looking at building automated retinal layer segmentation machine learning models for OCT imaging analysis over decade since OCT imaging was introduced [21, 31, 32]. Some of these models were applied efficaciously to predict textural properties for analysing the variation in the structure of the retinal tissue, segment retinal vessels and other retinal lesions [33]. The artificial intelligence based deep learning models were implemented to perform classification, image processing,and feature extraction. Moreover, machine learning based techniques such as Random forest, Bayesian classifier, Bag of words (BoW) were first presented to perform text categorization which was later adapted for image classification, histogram of oriented gradients(HOG), scale-invariant feature transform and many others [33–41]. Since the introduction of Deep learning, it has gained a lot of popularity because of the large dataset it can handle. With the introduction of convolutional neural network(CNN) which is a typical Deep learning technique, more complex analyses for example; pattern recognition, object detection, image processing etc., as computer vision have been implemented successfully in many recent publications [23, 42–49]. The CNN gained much attention because of its ability to handle very large dataset. The typical CNN is made up of multiple convolutional layers of artificial neurons, pooling layers, which are nonlinear in nature, fully connected layers and used to analyze visual imagery [25]. In CNN, when the input image is presented, the first layer extracts basic features and each layer generates multiple activation functions which the output is sent to the next layer. The next layer identifies more complex features which are also passed to the next available layer for even more complex features such as parts of objects or full objects etc. The final layer outputs a set of confidence scores based on the classification to indicate the likelihood of an image to belong to a particular class. The CNN always has an input layer, hidden layers and output layer such layers are always fully connected. The pooling layer of the CNN however has the responsibility to reduce the actual spatial size of the convoluted feature [50]. Once the dimension is reduced, the required memory size for the model and its computational power for processing the data is also reduced to improve translation invariance. The types of pooling are the max pooling, min pooling, sum pooling and average pooling. The max pooling ensures that the highest value of a pixel from the section of the image covered by the kernel is selected. The min pooling ensures that the lowest value of a pixel from the section of the image covered by the kernel is selected. The sum pooling considers summation of all the values from the section of the image covered by the kernel while the average pooling considers the average of the values from the section of the image covered by the kernel [51]. The pooled feature maps are usually flattened so each can be converted into column matrix to serve as input value for further computations. The kernel filter size is usually smaller than the presented image and therefore covers a section of the image. Its movement across the image is based on a stride value extracted from the feature map. If the stride value is large then the feature map is expected to be smaller in that case. Where the stride value is equal to 2 or greater will result in loss of some feature of the image. As a result different kernels are employed to generate different feature maps after which the ReLU activation function is applied after each convolution step to ensure nonlinearity and reduction in computational complexity [51, 52]. The CNN has the ability to extract more sophisticated features from a given training dataset which can be used to establish patterns for detecting future problems. This unique ability makes CNN very interesting area especially in analyzing, classifying and solving issues relating to retinal OCT imaging like layer Segmentation [53–55]. According to Karri et al., 2017 [56], the dynamic nature of the CNN allows for more new techniques such as transfer learning to be included in training small dataset which in their article was successfully implemented to classify OCT image with diabetic macular edema and dry age-related macular degeneration. Fang et al., 2019 [3] in their article designed a lesion-aware CNN technique for OCT image classification which they called the Lesion Detection Network (LDN). The designed model created a corresponding attention map to identify macular lesions by focusing on the salient areas for detailed information extraction. There were four retina OCT image class labels presented for classifications which were neovascularization (CNV), diabetic macular edema (DME), drusen, and normal. The study then compared the results such as accuracy, specificity, sensitivity and related statistical measures with other standard methods from deep learning approach which had also performed classifications on the same or similar dataset. According to Li et al. (2019) [57], a similar classification for retina OCT images which was also made up of four class labels was conducted using Residual Network (ResNet50) which is a CNN with 50 layers deep. The results from the model were very impressive as it achieved an accuracy of 97.3%, Sensitivity: 96.3%, Specificity: 98.5%.

Yanagihara et al. (2020) [58] employed graphical processing unit (GPU) computation to support their model using generative adversarial network (GAN) for classification and detection of retinal disease which also achieved a very promising accuracy. Another research conducted by Rajagopalan et al., 2021 [59] used CNN to perform retinal OCT image classification generated an accuracy of 97.01%, sensitivity of 93.43%, and specificity of 98.07%. Again, the conclusion indicated that the results outperformed all the existing models based on retina OCT image classification when they did comparison with other standard applied methods as at that time. A similar study conducted by Upadhyay et al., 2022 [60] that also employed CNN with a batch normalization layer to adopt coherent behavior also enhanced the retina OCT image classification accuracy to 97.19% for retina disease which the result was very impressive. The attention-based CNN has also been applied successfully for efficient classification of OCT images by other previous researchers [61–63].

According to Sabour et al., 2017 [30], the CNN has limitations which affect the performance of their models. In the article, some of the limitations identified were loss of important features of the image due to pooling operation, pixel perturbations, inability to recognize pose, texture or deformation etc. Hence the article proposed a Capsule Network(CapsNet) with a dynamic routing algorithm to address some of these challenges. The effectiveness of the capsNet necessitated many researchers into improving the routing-by-agreement algorithm. Zhang et al, (2018) proposed two fast routing algorithms after generalizing the existing algorithms to enhance the performance of the CapsNet [64]. This framework was implemented based on kernel density estimation. Also, Choi et al., (2019) also proposed attention routing between capsules to enhance the performance [65]. Other researchers also focused on applying capsNet to other forms of image classification [66]. However, it has received very little attention in the area of retina OCT image classification to help deal with diseases associated with the eye. A study presented by Santos et al., (2020) proposed a computational method for automatic classification of glaucoma which they deployed using Capsule Network [67]. The results indicated 90.90% accuracy, 86.88% recall, 94.64% precision, 90.59% f1-score, 0.904 AUC and 0.801 kappa index which is a very promising output since it did not require applying further data boost and segment the region of the optical disc. The study considered just glaucoma and not the other forms of eye diseases. However, the study also indicated that the capsule has a potential to establish a relationship between the characteristics of image even with respect to reduced set training. A similar paper also employed capsule network to segmentation subretinal fluid (SRF) from central serous chorioretinopathy (CSCR) whiles others focused on identifying the growth rate and level of spread through the various stages of the eye diseases [68].

Due to the potential performance of capsule network, this study employs the method for the classification of eye disease into CVN, DME, DRUSEN, and NORMAL using retina OCT images.

## 3 Proposed methods

The objective of the paper is to design a capsule network model with robust feature extractor to achieve high recognition accuracy on retina OCT image classification. We explore several model modifications and arrive at the following combinations for our model:

- CLAHE-CapsNet Architecture: The model was designed in a way to reduce the noise in the input image and extracts more textural features rather than image shapes and edges while at the same time reducing the number of trainable parameters.
- Power Squash: We adopt the power version ||*v*_*j*_||^*n*^ $\frac{v_{j}}{\left|\right|v_{j}\left|\right|}$ of the original squash function based on (Yang & Wang, 2019). It suppresses smaller activation values compared to larger ones (see Fig 3).
- Sigmoid Activation: In contrast to SoftMax, the Sigmoid activation function improves the distribution of coupling coefficients which leads to improved network performance. Experimental results show that the function improves model accuracy and convergence.

### 3.1 Capsule network

Deep learning-based CNN modes are widely used for feature extraction, detection, classification etc. The operations of the convolution in CNNs are simple in tackling complex problems [32]. Moreover, CNN does not consider the orientation of the components and relationship in space of features in an image but only cares about the presence of features. Sabour et al, 2017 proposed CapsNet to alleviate the aforementioned challenges of CNN and to represent a sample of visual entities. Capsules in the capsule network are collective neurons that show the activity vectors representing existing pose parameters. The length of the vector indicates the existence of an entity. One problem of CNNs is related to the pooling layers. Hence, capsule networks have replaced pooling layers with an algorithm called “routing by agreement.” Based on this method, the outputs from the lower layer are sent to all parent capsules in the higher layer. However, their coupling coefficients are different. Each capsule in the lower layer predicts the output of the parent capsules. If the prediction matches the parent capsule’s output, then the coupling coefficient for these two capsules is increased. Let *u*_*i*_ be the output of capsule i and its prediction from parent capsule *j* is expressed as

$$\begin{array}{c} s_{j}=\sum_{i}c_{j}{\hat{u}}_{j|i} \end{array}$$ (1)

A nonlinear function is used to shrink long and short vectors to 1 and 0 respectively. This is called a squash function meant to prevent the output vectors from exceeding 1. Eq (2) shows the non-linear squash function.

$$\begin{array}{c} v_{j}=\frac{\left|\right|s_{j}{\left|\right|}^{2}}{1+\left|\right|s_{j}{\left|\right|}^{2}}\frac{s_{j}}{\left|\right|s_{j}\left|\right|} \end{array}$$ (2)

where *s*_*j*_ in Eq (2) is the input vector to the *jth* capsule and *v*_*j*_ is the output vector. CapsNet adopts non-linearity squashing function on output vectors (*v*_*j*_) in each iteration [21]. This shows the likelihood of the vector between 0 and 1, which means that it squashes small vectors and maintains long vectors in the unit length

$$\begin{array}{c} v_{j}\approx \left|\right|s_{j}\left|\right|s_{j}=0\approx \frac{\left|\right|s_{j}\left|\right|}{\left|\right|s_{j}\left|\right|} \end{array}$$ (3)

The log probabilities are updated in the routing process based on the agreement between *v*_*j*_ for the fact that the agreement between two vectors will be increased and have a large inner product. Therefore, agreement *a*_*ij*_ for updating the log probability and coupling coefficient is defined as

$$\begin{array}{c} a_{ij}=v_{j}{\hat{u}}_{j|i} \end{array}$$ (4)

**Fig 1:**
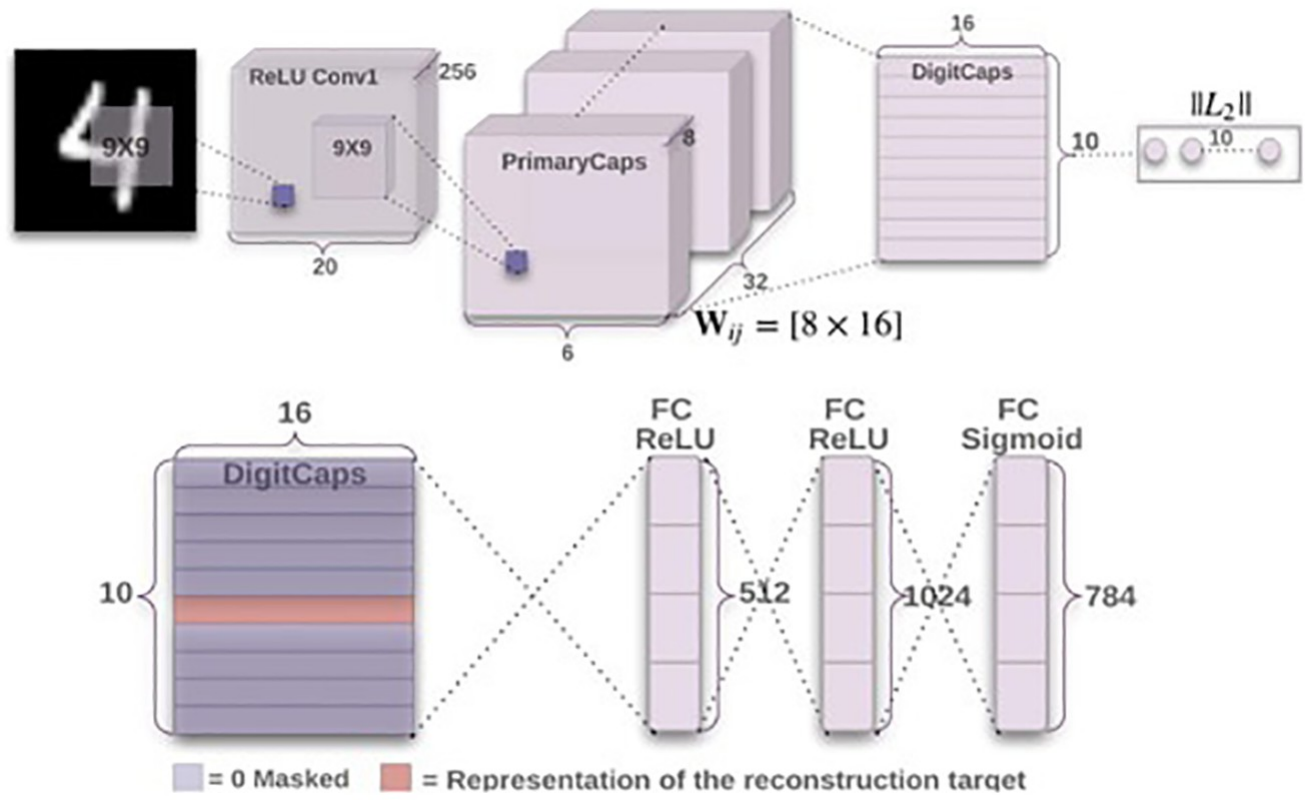
Original capsule network (CapsNet) by Sabour et. al., 2017.

Capsule *k* in the last layer is connected with a loss *l*_*k*_. This puts a big loss value on capsules with long output instantiation parameters when the entity does not exist. The loss function *l*_*k*_ is expressed as follows.

$$\begin{array}{c} l_{k}=T_{k}\;\text{max}\;{\left(0,m^{+}-\left|\right|v_{k}\left|\right|\right)}^{2}+\lambda (1-T_{k})\;\text{max}\;{\left(0,||v_{k}\left|\right|-m^{-}\right)}^{2} \end{array}$$ (5)

where *T*_*k*_ is 1 when class k is present, and is 0 otherwise. The m+, *m*^−^, and λ are hyperparameters that are set before the learning process. Fig 1 indicates the dynamic routing procedure.

### 3.2 CLAHE enhancement

The commonly adopted method in image enhancement often used is histogram equalization. This is due to its simplicity and low computation load. In this paper, contrast-limited adaptive histogram equalization (CLAHE) was used to improve the color and reduce the noise of the X-Ray images. CLAHE is an advanced form of adaptive histogram equalization (AHE) for image enhancement, which works well for biomedical images like MRI and mammograms [38]. It improves the image’s quality by removing the noise and preventing high noise amplification, resulting in the AHE technique. The method uses contrast amplification, limiting each neighboring pixel’s procedure, and the transformation function is formed to reduce the noise problem. Using the method manually as a pre-processing method, occupies additional space on the storage resource. In this study the CLAHE was implemented as an enhancement layer base before the convolution layer of the proposed model. The layer receives input from the initial input layer, processes it, and sends the output to the convolution layer.

### 3.3 Proposed architecture

**Fig 2:**
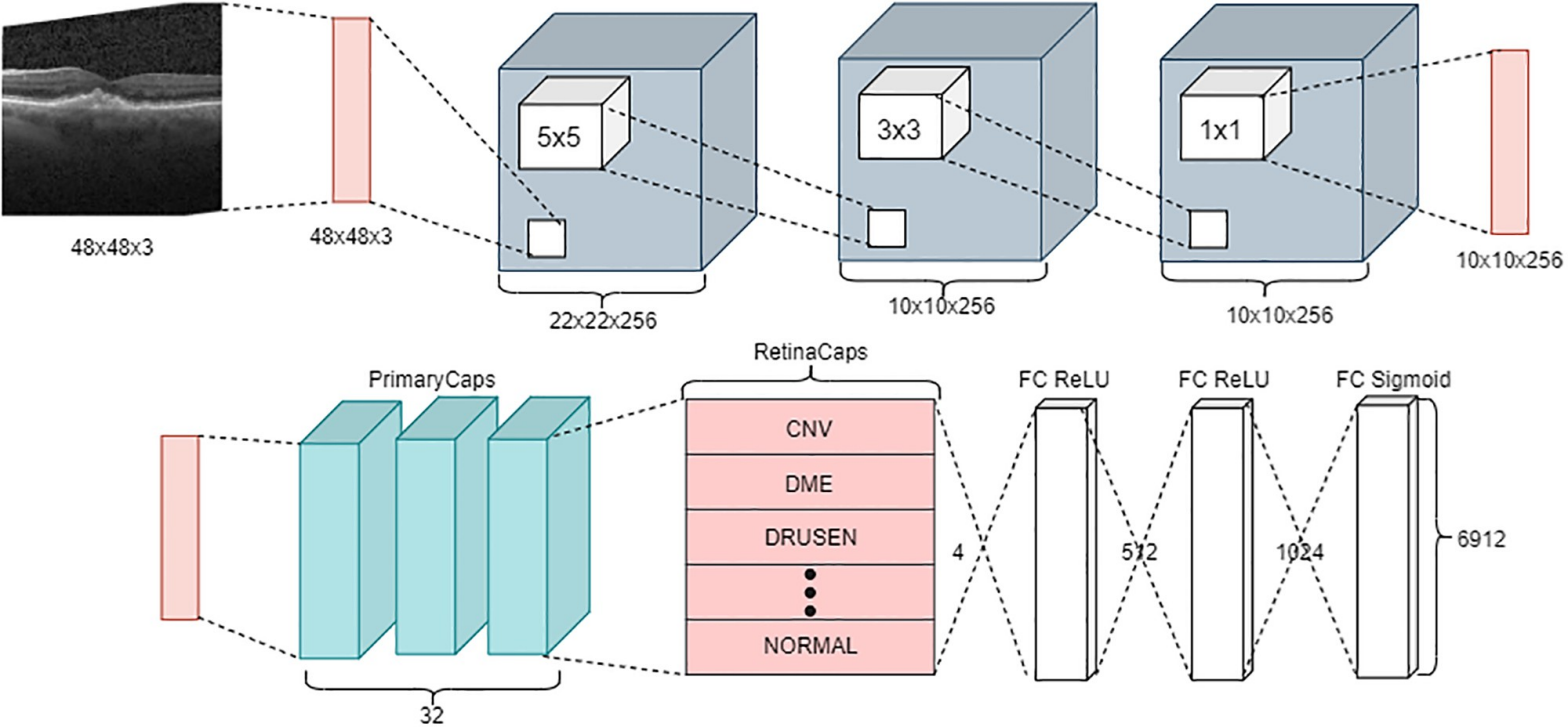
Proposed CLAHE-CapsNet architecture.

Fig 2 illustrates the proposed CLAHE-CapsNet architecture. The model is made up of CLAHE layers, Convolutional layers, a Primary Capsule (PC) layer and a classification layer (retinaCaps). The input images with the dimension of 48 × 48 × 3 are fed to the initial Clahe layer (Clahe1). The layer is an enhancement layer and therefore does contribute additional parameters to the model. The layer gives output feature size of 48 × 48 × 3 and is supplied to the Conv1. The Conv1 with kernel = 5 × 5, and Stride of 2, receives the feature map produce and output feature map 22 × 22 × 256. The output is fed into Conv2 with kernel = 3 × 3 and Stride of 2 that produces feature maps 10 × 10 × 256, and is sent to Conv3 with kernel = 1 × 1 and Stride to output feature map of 10 × 10 × 256. Again, the output from the third convolution is fed to Clahe2 for additional noise reduction. ReLU activation function used in the convolutional layers. The Clahe2 give the same feature size of 10 × 10 × 256 and it is fed into the PC layer. The PC layer consists of convolutional capsule layer with kernel = 9 × 9 and stride of 3. At the PC layer, a tensor product between u and the weights (W) produces ${\hat{u}}_{j|i}$ made up of 576 (i.e., 4 × 4 × 16), 8-dimensional vectors. At the Digit Caps layer. the Recognition Caps will form *k*, 16D vectors, where *k* = number of classes. There are three Fully connected (FC) layers in the decoder network consisting of 512, 1024, and 6912 neurons in the first, second, and third layers respectively.

#### 3.3.1 Power squash function

**Fig 3:**
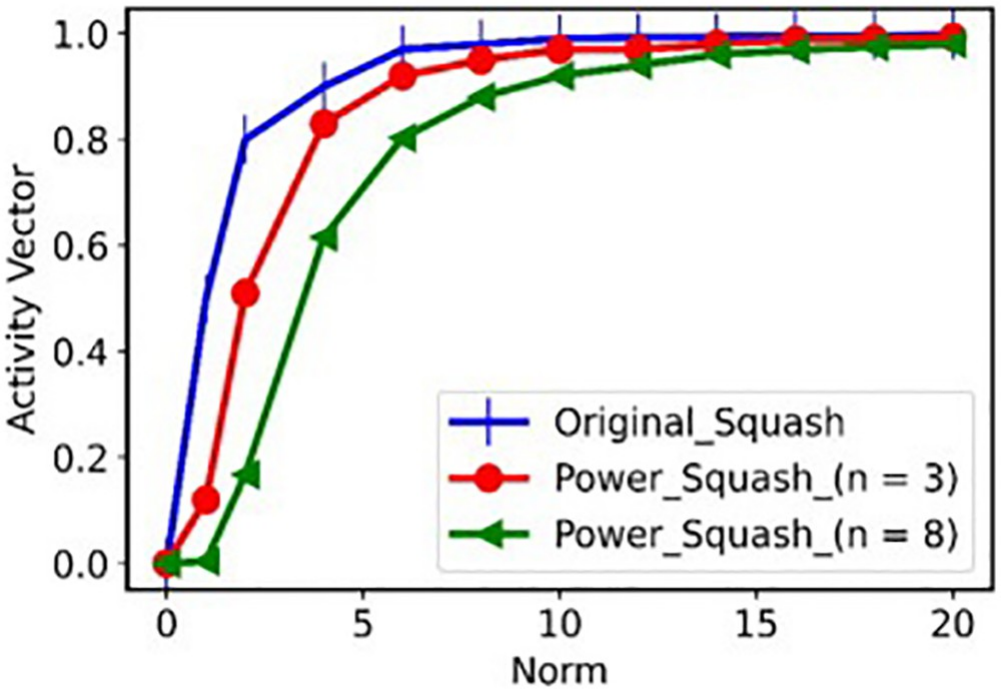
Comparison of original squash and power squash activation function.

This paper uses the power squash proposed by Yang and Wang, [69] to maintain the direction input vectors *s*_*j*_ and also to compress the length within the range [0, 1]. The purpose of compressing is to make sure short vectors are compressed to almost zero (0) length whiles the long vectors are compressed slightly below one (1). The function $\frac{\left|\right|s_{j}{\left|\right|}^{2}}{1+\left|\right|S_{j}{\left|\right|}^{2}}$ used in the original squash function indicates the scales with $\frac{S_{j}}{\left|\right|S_{j}\left|\right|}$ which represents the unit vectors *s*_*j*_. Although this function is promising in the original CapsNet, however, it has difficulty in alleviating the high information sensitivity which leads to abnormally high activation values distribution of capsules in the primary capsule (PC) layer (Zonglin & Wang, 2019). This squash was chosen for the proposed model due to the high activation values for smaller ||*s*_*j*_|| which results in faster growth of the function. Fig 3 shows a comparison of power squash and the original squash functions. The figure proves that there is faster-initial growth in the original squash function than the power function. This shows that the function does not provide sparsity needed to constrain the capsules from obtaining high activation values.

## 4 Experimental result

### 4.1 Dataset

**Fig 4:**
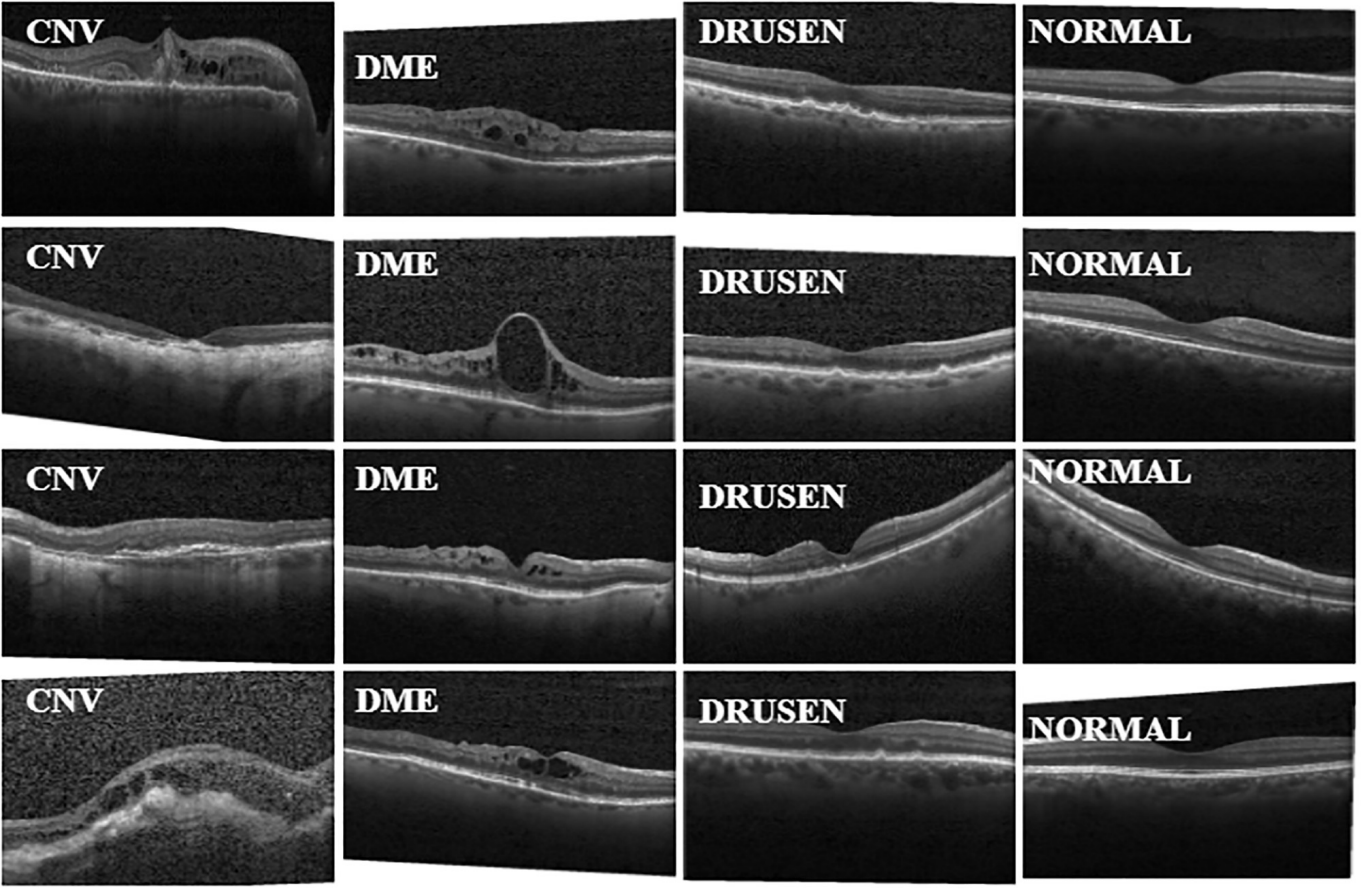
Sample Retina OCT images.

The dataset used in the paper is organized into 3 folders (train, test, val) and was downloaded from Kaggle.com (https://www.kaggle.com/paultimothymooney/kermany2018, accessed on January 9, 2023). The Folders contain subfolders for each category of the image, i.e. NORMAL, CNV, DME, and DRUSEN. The dataset consists of 84,495 X-Ray images (JPEG) and 4 categories (NORMAL, CNV, DME, and DRUSEN). The images are labeled as (disease)-(randomized patient ID)-(image number by this patient) and split into 4 directories: CNV, DME, DRUSEN, and NORMAL. Optical coherence tomography (OCT) images were selected from retrospective cohorts of adult patients from institutions such as Shiley Eye Institute at University of California San Diego, California Retinal Research Foundation, Medical Center Ophthalmology Associates, Shanghai First People’s Hospital, and Beijing Tongren Eye Center. The selection was done between July 1, 2013, and March 1, 2017. The images went through a grading system consisting of multiple layers of trained graders of expertise for verification and correction of image labels. Images imported into the database started with a label matching the recent diagnosis of the patient. The first group of graders consisted of undergraduate and medical students who had passed an OCT interpretation course review. These first graders performed initial quality control and excluded OCT images containing critical artifacts or significant image resolution reductions. Secondly, four ophthalmologists who were second graders independently graded the image that had passed the first grading. choroidal neovascularization, macular edema, drusen, and other pathologies which are present or absent on the OCT scan were recorded. Lastly, the third group of graders consisted of two senior independent retinal specialists. Each specialist has over 20 years of clinical retinal experience, who varied the true label of the images. The sample dataset selection is illustrated in a CONSORT-style Fig 4. The total dataset was 84,495 images, however, we observed there was an unbalanced dataset which can lead to high misclassification where the model will predict the images for the instance with the high images. Therefore, in this study, the dataset was subsampled to 36,496 images. Table 1 presents a description of the dataset. In Table 1, it can be observed that the dataset was splitted into Train set, test set and validation set which consisted of 8616 for the CNV, DRUSEN, and DME except the Normal instance which consisted of 8712, respectively for the train set. However the test and validation consisted of 242 images, respectively.

**Table 1:** Dataset description.

Classes	Train set	Test set	Validation set	Total
CNV	8,616	242	242	9,100
DME	8,616	242	242	9,100
DRUSEN	8,616	242	242	9,100
NORMAL	8,712	242	242	9,196
**Total**	**34,560**	**968**	**968**	**36,496**

### 4.2 Experimental settings

This paper’s experiments were implemented using a Windows system with NVIDIA GeForce GTX 1650 6GB GPU. The codes take TensorFlow as the backend and are implemented through Keras and python (Anaconda). The network was trained for 100 epochs on the proposed and original CapsNet models, respectively. The learning rate was set to 0.0001, and the batch size on the original images was set to 32. We used the Adam algorithm with momentum as the gradient optimizer. The momentum was set to 0.9, and the descent rate was set to 10–6. The code used for the study is a modified code which is available at https://github.com/XifengGuo/CapsNet-Keras.

## 5 Results

This section presents the result of the proposed model and comparison with the original CapsNet by Sabour et al., 2017. Additionally, a comparison of results was done with other state-of-the-art (SOTA) models used model for classifying the retina OCT dataset. Result comparison is done to determine the best model for retina OCT classification. Evaluation models such as accuracy (ACC), sensitivity (SE), precision (PR), specificity (SP), receiver operating characteristic-area under ROC curve (ROC-AUC), and confusion matrix are used to determine the performance of the models. To avoid the imbalance of samples among different classes, overall accuracy (OA), overall sensitivity (OS), and overall precision (OP) are also computed. Overall accuracy (OA) is evaluated in Eq 6.

$$\begin{array}{c} OA=\frac{\text{correct}\;\text{classified}\;\text{sample}}{\text{total}\;\text{number}\;\text{of}\;\text{samples}} \end{array}$$ (6)

ACC stands for accuracy which is the overall correctness of the model’s predictions. SE (Sensitivity) is also known as recall or true positive rate, measures the proportion of actual positives that are correctly identified as positive by the model. PR (Precision) measures the proportion of true positives out of all the positives predicted by the model. SP (Specificity) measures the proportion of actual negatives that are correctly identified as negative by the model. AUC (Area Under the ROC Curve) is a measure of the overall performance of the model. OA (Overall Accuracy) is the accuracy of the model for all classes combined. OS (Overall Sensitivity) is the sensitivity of the model for all classes combined. OP (Overall Precision) is the precision of the model for all classes combined.

**Fig 5:**
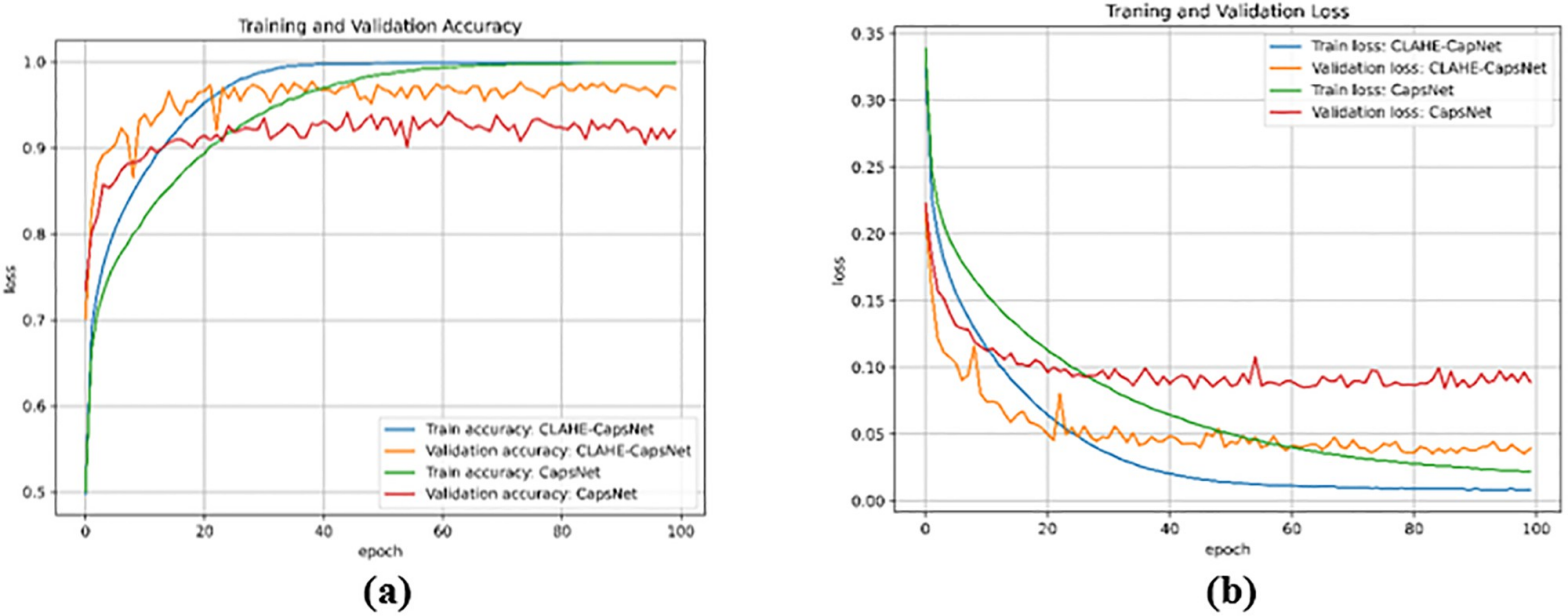
Training, validation accuracy and loss curve on Retina OCT images. (a) Training and validation accuracy curves of CLAHE-CapsNet and original CapsNet., and (b) Training and validation loss curves of CLAHE-CapsNet and original CapsNet.

**Fig 6:**
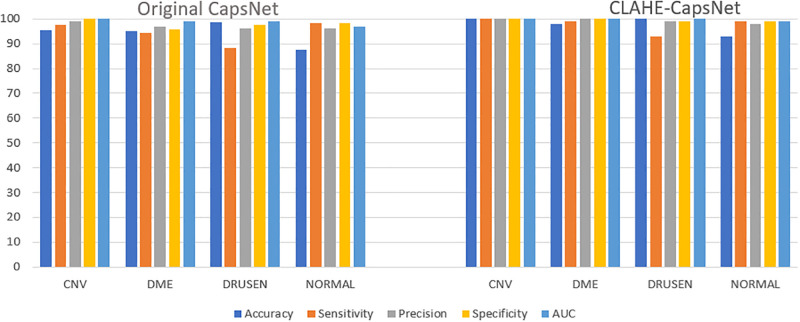
Performance comparison of model based on individual classes (i.e., CNV, DME, DRUSEN, and NORMAL).

**Fig 7:**
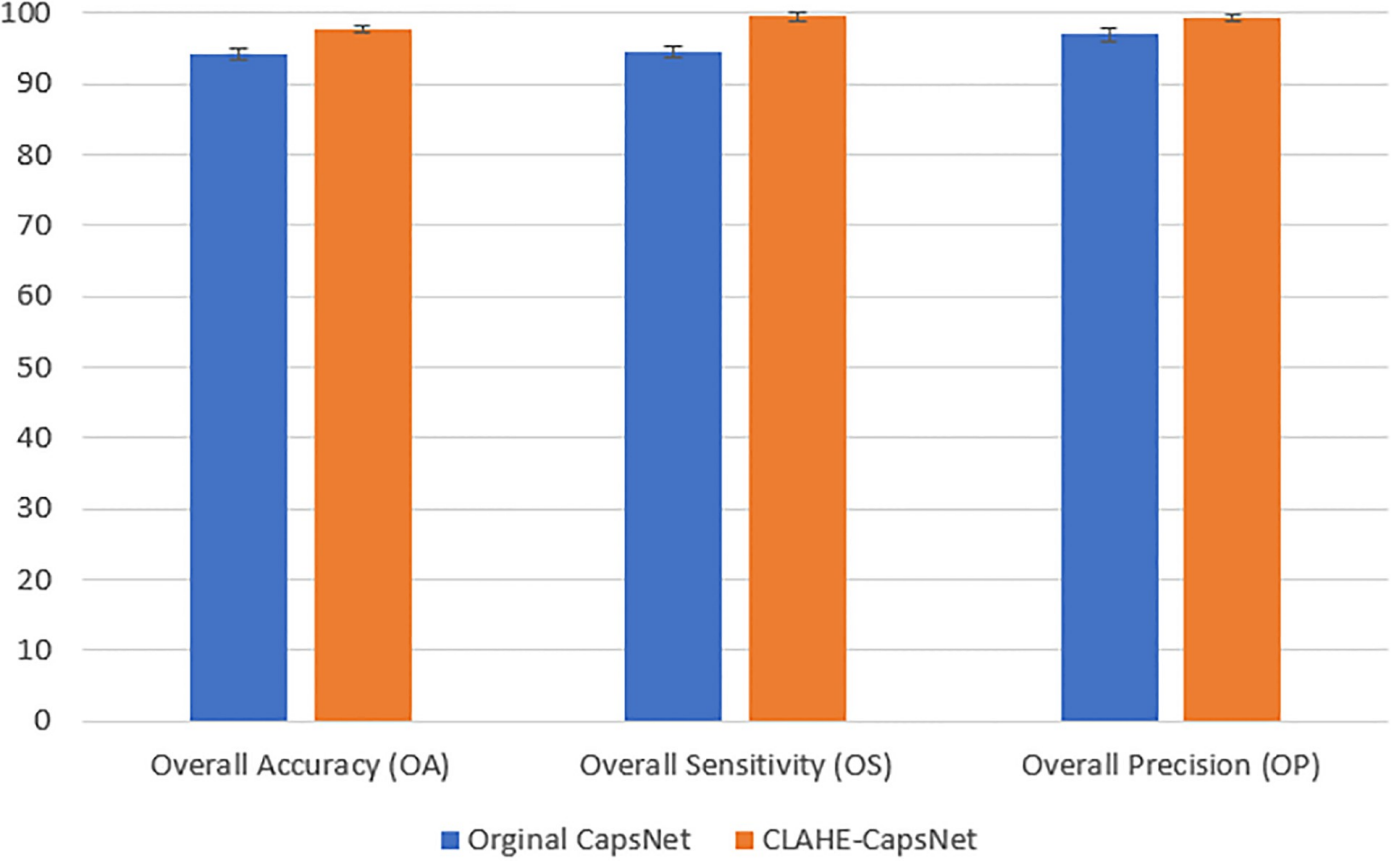
Histogram accuracy comparison based on the overall accuracies of OA, OS, and OP.

Table 2 presents a results comparison of CLAHE-CapsNet and the original capsNet architecture. CLAHE-CapsNet achieved the highest accuracies of 97.7%, 99.5%, and 99.3%, respectively on OA, OS, and OP compared to the original CapsNet of 94.2%, 94.5%, and 97.0%. Our model achieved the best performance in all rounds of the evaluation metrics than the original CapsNet except the AUC of the CNV instance where both models obtained the same accuracy of 100%. Fig 5(a) illustrates the training and validation accuracy comparison of CLAHE-CapsNet and original CapsNet. It can be observed from the curves that the proposed model outperformed the original CapsNet in both the training and validation accuracies. Fig 5(b) shows the training and validation loss of the models. The proposed model shows good performance by obtaining the least losses on the training and validation. Fig 6 shows a histogram of accuracy comparison of the CLAHE-CapsNet and the original CapsNet based on the four instances of the dataset (CNV, DME, DRUSEN, and NORMAL). A comparison of result with OA, OS, and OP based on the orginal CapsNet and CLAHE-CapsNet is shown in Fig 7.

**Table 2:** Comparison of results of the proposed model and original CapsNet. The best performance is labeled in **bold**.

Method	Classes	ACC (%)	SE (%)	PR (%)	SP (%)	AUC (%)	OA (%)	OS (%)	OP (%)
Original CapsNet [30]	CNV	95.5	97.5	99.0	100	100	94.2	94.5	97.0
	DME	95.0	94.3	97.0	95.8	99			
	DRUSEN	98.8	88.2	96.0	97.6	99			
	NORMAL	87.6	98.2	96.0	98.2	97			
CLAHE-CapsNet [Ours]	CNV	**100.0**	**100**	**100**	**100**	**100**	**97.7**	**99.5**	**99.3**
	DME	**97.9**	**99.0**	**100**	**100**	**100**			
	DRUSEN	**100.0**	**93.0**	**99.0**	**99.0**	**100**			
	NORMAL	**93.0**	**99.0**	**98.0**	**99.0**	**99.0**			

**Fig 8:**
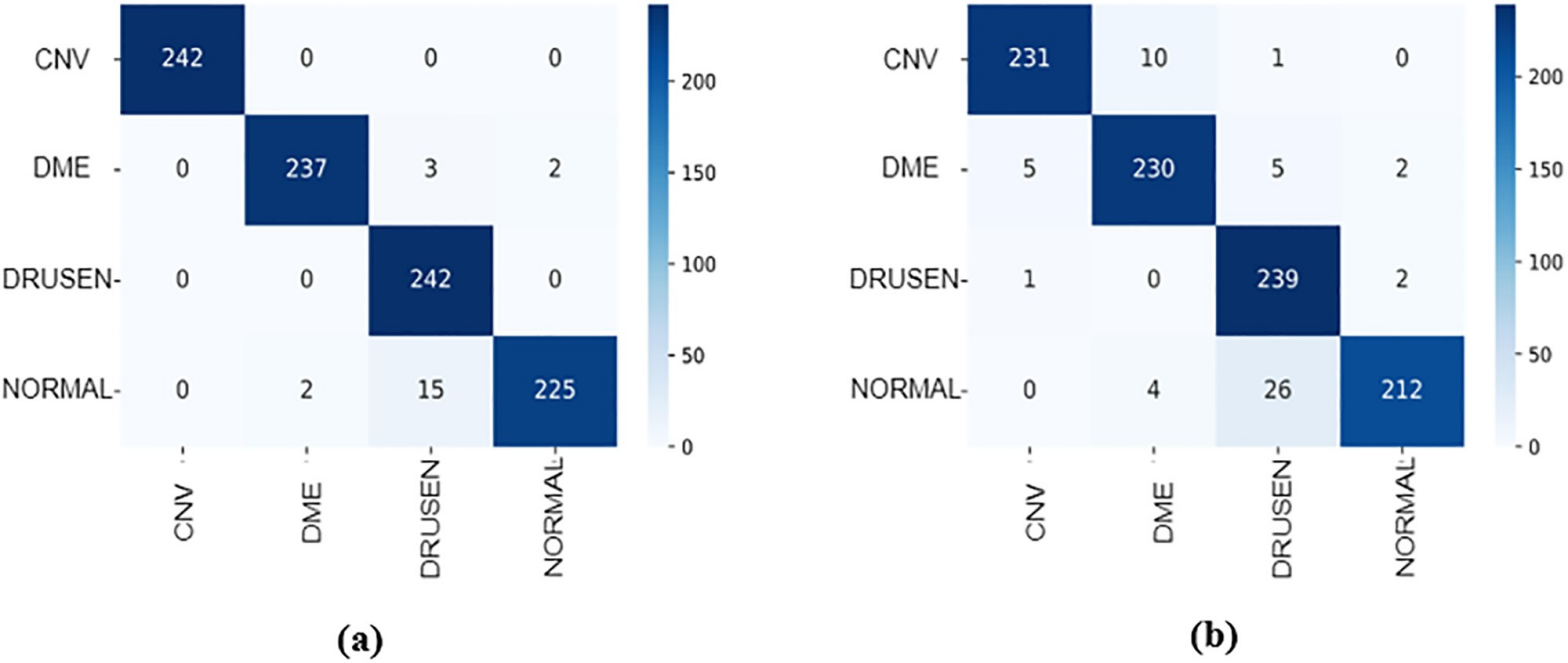
Confusion matrix comparison on the proposed model and original CapsNet.

**Fig 9:**
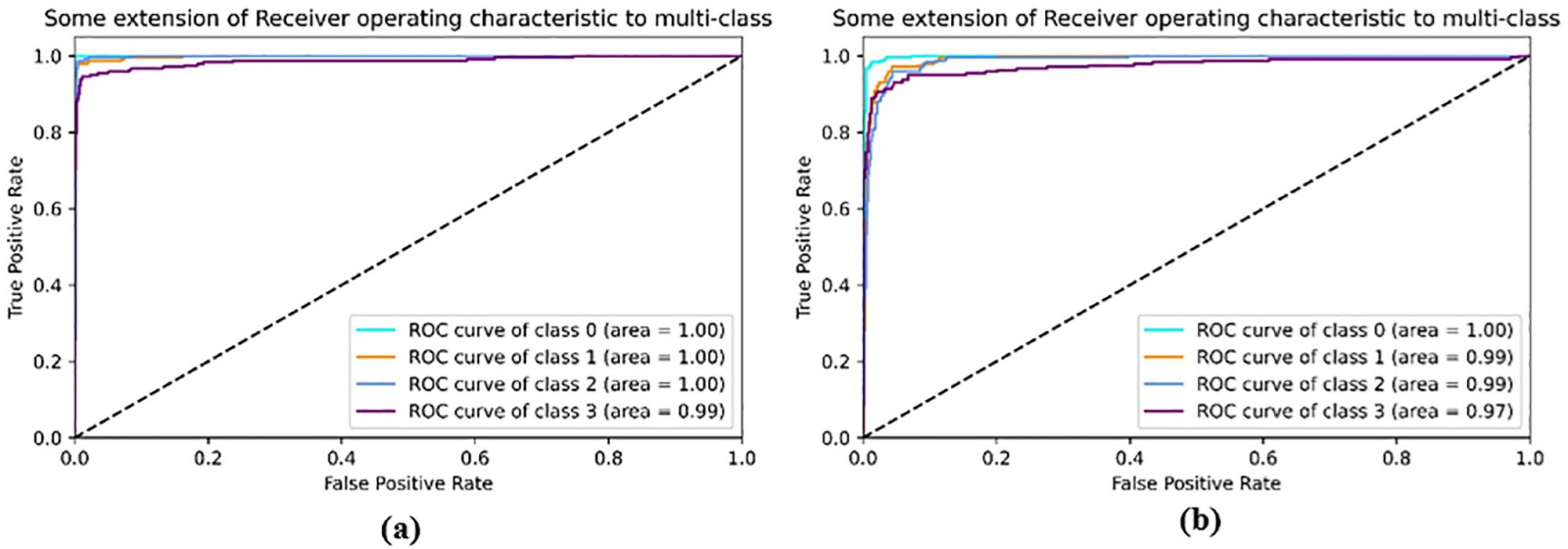
Comparison of ROC-AUC on the proposed model and original CapsNet. (a) CLAHE-CapsNet ROC curve, and (b) Original CapsNet ROC curve.

**Fig 10:**
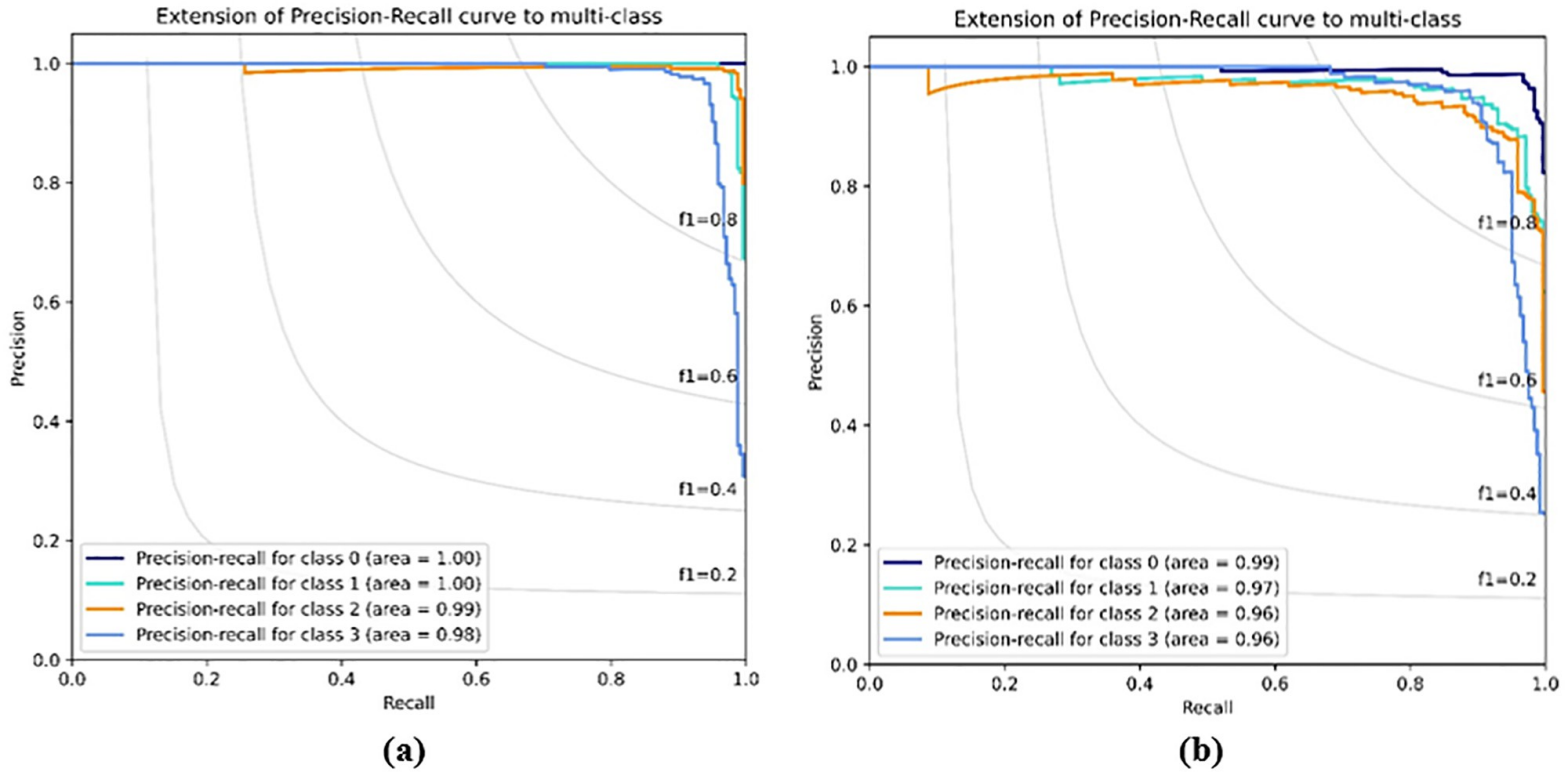
Precision-Recall curves comparison on the proposed model and original CapsNet. (a) CLAHE-CapsNet Precision-Recall curve, and (b) Original CapsNet Precision-Recall curve.

Fig 8(a) and 8(b) show the confusion matrix of the evaluated capsNet models. The diagonal outputs (blue) illustrated the correct prediction from the models (True positives and True Negatives). The output at the upper and bottom part of the correct prediction (white) are misclassifications generated from the model. Hence, it shows the models wrongful classification to other classes. Fig 8(a) presents the confusion matrix from the proposed model in this work. Whereas Fig 8(b) presents the confusion matrix from the original CapsNet. The proposed method achieved the high correct prediction of 242, 237, 242, and 225 on CNV, DME, DRUSEN, and NORMAL, respectively, compared to the original capsNet performance of 231, 230, 239, and 212. Based on the confusion matrix the CLAHE-CapsNet model recorded the least misclassification than the original capsNet. This can be attributed to the fact that the original CapsNet model has only one (1) convolutional layer that fails to extract enough and proper features in the images. Fig 9 illustrates the ROC-AUC curves and Fig 10 shows the Precision-Recall curve. Based on the result that can be observed from Figs 9 and 10, the proposed model better handles misclassification than the original capsule.

**Fig 11:**
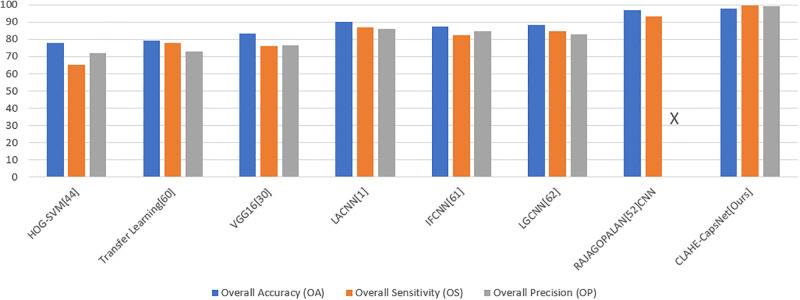
Comparison of results with state-of-the-art works based on the individual classes.

**Fig 12:**
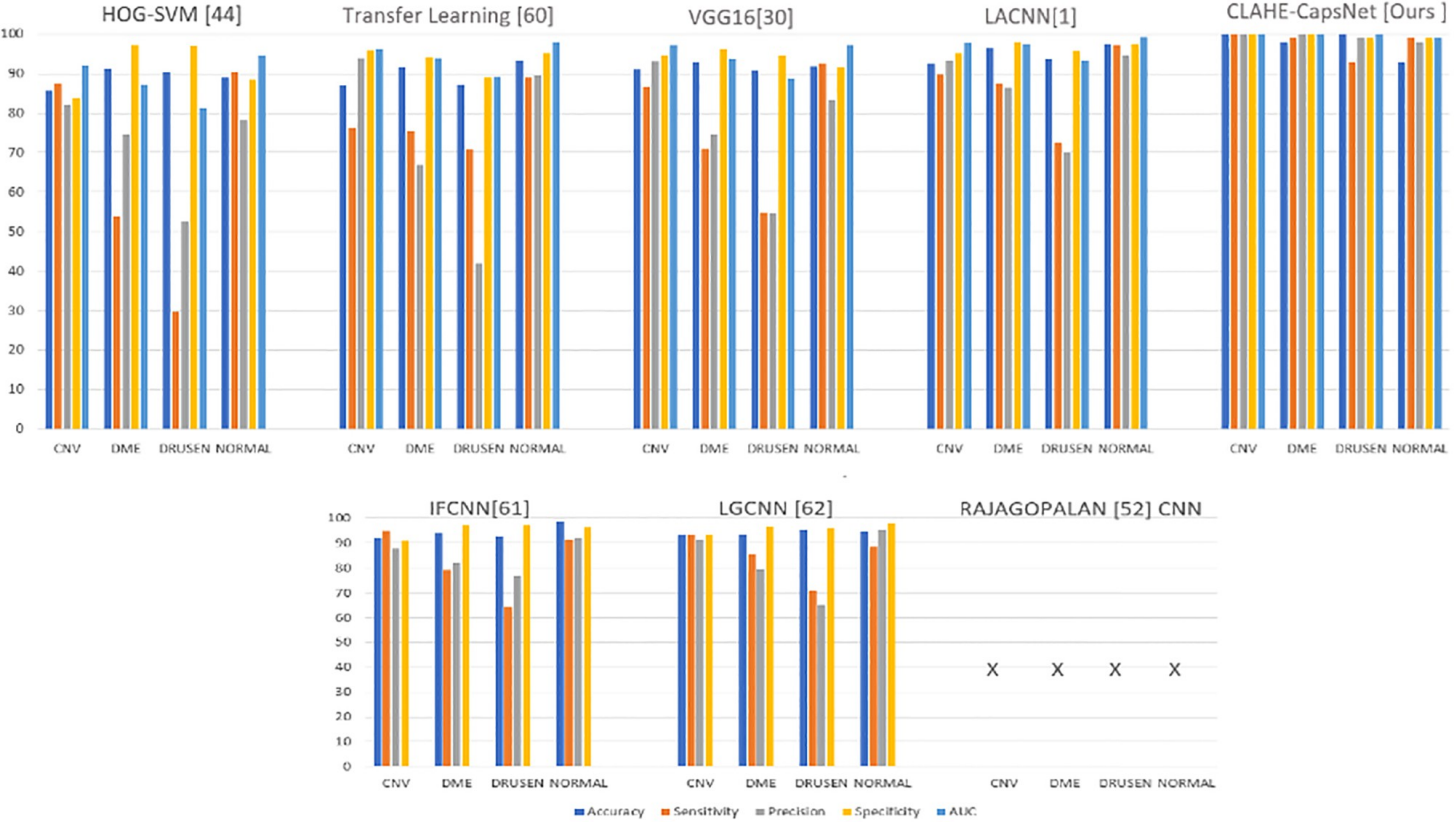
Comparison of results with state-of-the-art works based on the individual classes.

To further evaluate the performance of the proposed model, a comparison of results with other state-of-the-art results that used the same dataset was conducted based on accuracy, sensitivity, precision, specificity, overall accuracy, overall sensitivity, and overall precision evaluation metrics. Table 3 shows results of comparison of the CLAHE-CapsNet and previous works. The comparison of the result was based on performance of the individual classes of the retina OCT dataset. The letter “x” indicates that the work did not report the result for the particular evaluation metric. Our proposed model recorded the highest performance on all the classes using the ACC, SE, PR, SP, and AUC evaluation metrics. It can be observed from Table 3 that CLAHE-CapsNet obtained OA, OS, and OP results of 97.9%, 99.5%, and 99.3%, respectively. Rajagopalan [59] using CNN achieved the second-best result of 97.0%, and 93.4%, on OA and OS. Their paper failed to report the result for OP. From Table 3, it can be observed that our model outperformed the other works compared. Leyuan et. al., 2019 [3] architecture named Lesion Attention Convolutional Neural Network (LACNN) managed to obtain the third best model with accuracies of 90.1%, 86.8%, and 86.3% on OA, OS, and OP, respectively. The HOG-SVM model achieved the least performance of 78.1%, 65.3%, and 71.8% on OA, OS, and OP, respectively. Fig 11 shows Histogram accuracy comparison based on the overall accuracies of OA, OS, and OP for HOG-SVM, Transfer Learning, VGG16, LACNN, IFCNN, LGCNN, CNN by Rajagopalan, and CLAHE-CapsNet. The figure shows that our model outperformed the other models in all the metrics used. Finally, Fig 12. Compares the results of our model with state-of-the-art works based on the individual classes. The performance of the proposed model can be attributed to the fact that the CLAHE layers adequately reduce the noise in the input images and the CapsNet with the dynamic routing algorithm is able to recognize the pose, texture and the deformation in the images whereas the recognition of pose, texture, and deformation is a limitation to CNN models.

**Table 3:** Comparison of results of the proposed model and other state-of-the-art works. The best performance is labeled in **bold**.

Method	Classes	ACC (%)	SE (%)	PR (%)	SP (%)	AUC (%)	OA (%)	OS (%)	OP (%)
HOG-SVM [23]	CNV	85.7	87.6	82.0	84.0	92.2	78.1	65.3	71.8
	DME	91.4	53.8	74.6	97.2	87.3			
	DRUSEN	90.2	29.5	52.6	97.0	81.3			
	NORMAL	89.1	90.4	78.1	88.4	94.6			
Transfer Learning [70]	CNV	86.9	76.2	93.9	95.9	96.1	79.5	77.9	73.1
	DME	91.6	75.5	66.9	94.1	93.8			
	DRUSEN	87.2	70.7	42.0	89.1	89.2			
	NORMAL	93.3	88.9	89.5	95.2	98.1			
VGG16 [43]	CNV	91.0	86.6	93.2	94.7	97.2	83.2	76.2	76.4
	DME	92.8	70.9	74.6	96.2	93.6			
	DRUSEN	90.7	54.7	54.5	94.7	88.7			
	NORMAL	91.8	92.6	83.3	91.5	97.2			
LACNN [3]	CNV	92.7	89.8	93.5	95.1	97.7	90.1	86.8	86.3
	DME	96.6	87.5	86.4	98.0	97.4			
	DRUSEN	93.6	72.5	70.0	95.6	93.4			
	NORMAL	**97.4**	97.3	94.8	97.4	99.2			
IFCNN [71]	CNV	92.4	94.8	87.9	90.9	X	87.3	82.5	84.7
	DME	94.4	79.2	81.9	97.2	X			
	DRUSEN	93.0	64.4	76.8	97.3	X			
	NORMAL	98.4	91.5	92.2	96.4	X			
LGCNN [72]	CNV	93.3	93.3	91.5	93.3	X	88.4	84.6	82.9
	DME	93.6	85.7	79.4	96.8	X			
	DRUSEN	95.4	71.0	65.2	96.0	X			
	NORMAL	94.6	88.5	95.5	97.9	X			
Rajagopalan et. al., [59] CNN	CNV	X	X	X	X	X	97.0	93.4	X
	DME	X	X	X	X	X			
	DRUSEN	X	X	X	X	X			
	NORMAL	X	X	X	X	X			
CLAHE-CapsNet [Ours]	CNV	**100.0**	**100**	**100**	**100**	**100**	**97.7**	**99.5**	**99.3**
	DME	**97.9**	**99.0**	**100**	**100**	**100**			
	DRUSEN	**100.0**	**93.0**	**99.0**	**99.0**	**100**			
	NORMAL	93.0	**99.0**	**98.0**	**99.0**	**99.0**			

### 5.1 Discussion

The study evaluated the proposed method (CLAHE-CapsNet) and compared the result to the original CapsNet by Sabour. Table 2 presented an analysis of the performance metrics for two different methods (Original CapsNet and CLAHE-CapsNet) on a task that involves classifying different classes (CNV, DME, DRUSEN, NORMAL). The performance metrics reported include accuracy (ACC), sensitivity (SE), precision (PR), specificity (SP), area under the curve (AUC), overall accuracy (OA), overall sensitivity (OS), and overall precision (OP). For each class, Table 2 reports the performance metrics for both methods. Based on Table 2, it appears that the CLAHE-CapsNet method outperforms the Original CapsNet method on several metrics, particularly in the DME and DRUSEN classes. For example, in the DME class, the CLAHE-CapsNet method achieves higher accuracy, sensitivity, and AUC compared to the Original CapsNet method. However, in the NORMAL class, the Original CapsNet method achieves higher accuracy, sensitivity, and AUC compared to the CLAHE-CapsNet method. The performance from the proposed method can be attributed to the increase in the number convolutional layers and contrast limited adaptive histogram equalization for image enhancement to the proposed method. The original CapsNet has only one (1) convolutional layer, which is not enough to extract more features and hence, this limits the performance of the mode. In contrast, the proposed model has three (3) convolutional layers and a layer to improve the image quality. This technique helped in extracting more and appropriate features that contribute to achieving the high accuracy compared to the original CapsNet.

In Table 3, different models have been evaluated on various classes such as CNV, DME, DRUSEN, and NORMAL. It appears that the results show that CLAHE-CapsNet has the best performance on all metrics, with 100% accuracy for CNV and DRUSEN classes, and high accuracy for DME and NORMAL classes as well. Other models have also performed well on different classes and different metrics. The other methods used the CNN architecture for feature extracting and prediction. However, CNN’s pooling operation is a limitation to the model due to its contribution to the reduction in loss of features. Meanwhile, the proposed model adopted capsule network architecture. In the capsule network the operation of pooling is not performed and this retains more features for better prediction performance. Moreover, the proposed model (CLAHE-CapsNet) adopted a contrast limited adaptive histogram equalization technique, which improves the equality of the input images. These techniques used in this study contributed to high performance accuracy compared to the other methods. It’s important to note that the performance of a model can vary depending on the specific dataset and the evaluation metrics used. Therefore, it’s crucial to select the appropriate evaluation metrics and to carefully evaluate the model on a representative dataset.

## 6 Conclusion

This paper presented a comprehensive literature review on current-state-of-art works on retina OCT image classifications. Moreover, a capsule network with contrast limited adaptive histogram equalization (CLAHE-CapsNet) for retina OCT image classification was proposed. To alleviate the abnormal activation values in the capsule by the original squash function we used power squash activation function. Four-class (CNV, DME, DRUSEN, and NORMAL) retina OCT image dataset presented by UCSD was used for training and testing the proposed capsule framework. Evaluation of models were conducted using evaluation metrics such ACC, SE, PR, SP, AUC on the individual classes while OA, OS, and OP were used to measure the overall performance of the models. The study compares the proposed model with the original capsule network for performance efficiency. The evaluation compares the proposed model with original CapsNet, and other state-of-the-art deep learning-based convolutional neural networks. The proposed model (CLAHE-CapsNet) achieved the best performance 97.7%, 99.5%, 99.3% on OA, OS, and OP, respectively than the other works. This performance indicates that the proposed technique is better in detecting eye diseases from retina OCT images. The method can be adopted to help ophthalmologists in detecting eye disease from retina OCT images. Limitation of the proposed model is the less compression ability of the CNN layers. In our future work, Fourier Transform technique will be used to resolve the challenge of the CNN layer and provide better image filtering, and image.

## Abbreviations

ACC: Accuracy
AHE: Adaptive histogram equalization
AMD: Age-related macular degeneration
BoW: Bag of words
CapsNet: Capsule network
CLAHE: Contrast limited adaptive histogram equalization
CNN: Convolutional neural network
CNV: Choroidal neovascularization
CSCR: Central serous chorioetinopathy
DME: Diabetic macular edema
GAN: Generative adverserial network
GPU: Graphical processing unit
LACNN: Lesion attention convolutional neural network
LDN: Lesion detection network
MD: Macular degeneration
OA: Overall accuracy
OCT: Optical coherence tomography
OP: Overall precision
OS: Overall sensitivity
PC: Primary capsules
PR: Precision
ROC-AUC: Receiver operating characteristic-area under ROC curve
RPE: retinal pigment epithelium
SE: Senstivity
SOTA: State-of-the-art
SP: Specificity
SRF: Segmented subretinal fluid
UCSD: University of california san diego
WHO: World health organization
